# Parameter- and Compute-Efficient Spatial–Spectral Transformer Framework for Pixel-Level Classification of Foreign Plastic Objects on Broiler Meat Using NIR–Hyperspectral Imaging

**DOI:** 10.3390/s26082459

**Published:** 2026-04-16

**Authors:** Zirak Khan, Seung-Chul Yoon, Suchendra M. Bhandarkar

**Affiliations:** 1 School of Computing, University of Georgia, Athens, GA 30602, USA; zirak.khan@uga.edu (Z.K.); suchi@uga.edu (S.M.B.); 2 Quality and Safety Research Unit, U.S. National Poultry Research Center, U.S. Department of Agriculture—Agricultural Research Service, Athens, GA 30605, USA

**Keywords:** hyperspectral imaging, foreign object detection, foreign plastic objects, pixel-wise classification, transformer, efficient attention, computational efficiency, food safety, real-time inspection, sensor technology

## Abstract

Foreign plastic objects (FPOs) in poultry products present significant food safety risks and cause economic losses for the industry. Conventional detection methods, including X-rays and color imaging, often struggle to identify small or low-density plastics. Hyperspectral imaging (HSI) offers both spatial and spectral information but suffers from high computational cost when applied for FPO identification in industrial environments. This study introduces a parameter-efficient and computationally efficient spatial–spectral transformer framework for pixel-level classification of FPOs on broiler meat using NIR-HSI (1000–1700 nm). The framework integrates three innovations: (1) center-focused linear attention (CFLA) to reduce computational complexity from $O(n^{2})$ to O(n); (2) patch-local mixed-axis 2D rotary position embedding to preserve geometric relationships within hyperspectral patches; and (3) low-rank factorized projection (LRP) matrices to reduce parameters by approximately 50% within projection weight matrices. The framework was trained and evaluated on a dataset of 52 chicken fillets, comprising 295,340 labeled target hyperspectral pixels from 12 common polymer types and 1 fillet class. The model achieved 99.39% overall accuracy, 99.57% average accuracy, and a 99.31 Kappa coefficient across 248,540 test pixels. Per-class precision, recall, and F1-score exceeded 98.05%, 98.59%, and 98.76%, respectively, across all classes. Efficiency analyses showed an 83% reduction in multiply–accumulate operations (MACs), a 22% reduction in trainable parameters, and a model size reduction from 1.72 MB to 1.35 MB relative to the baseline configuration. These gains also translated into practical inference benefits, with the final model achieving a throughput of 212,971.5 hyperspectral patch cubes/s and a 4.19× speedup over the baseline. These results demonstrate that the proposed framework combines strong classification performance with high efficiency, supporting high-throughput inference for real-time monitoring and enabling contamination source traceability and preventive quality control in industrial poultry processing. The approach provides a benchmark for applying transformer-based models to food safety inspection tasks.

## 1. Introduction

Ensuring the quality and safety of poultry products remains a crucial concern for the poultry industry, as contamination by foreign materials (FMs) poses major health risks to consumers and can also lead to substantial economic losses through product recalls and reputational damage [1]. Non-biological FMs, such as plastic, rubber, and metal fragments, are among the most common contaminants and are often introduced unintentionally during processing stages, including portioning, trimming, deboning, and packaging [2,3]. Among these FMs, small foreign plastic objects (FPOs) have become an increasingly critical challenge in food safety. FPOs, often targeted for detection at approximately 2 mm or larger, are difficult to identify and may enter the food chain through various pathways, including degradation of packaging materials, wear of food processing equipment, and contamination during handling and transportation [4,5]. Although plastic fragments smaller than 5 mm are classified as microplastics in some food and environmental studies [6,7], in the context of food safety and industrial food processing, these particles are more appropriately considered FPOs, reflecting their origin as well as the detection and mitigation requirements within food processing environments.

The significance of FPO contamination extends beyond immediate food safety concerns. These plastic fragments not only pose physical hazards to consumers but also create complex challenges for food manufacturers in terms of detection, classification, and source identification. The public health implications of FPO ingestion are multifaceted, as these objects can act as physical contaminants while also containing or adsorbing hazardous chemicals, thereby increasing their potential toxicity. Research has linked exposure to certain polymer types with adverse health effects, including reproductive complications and impairments of the nervous and immune systems in both humans and animals [8]. However, the most critical aspect of FPO classification lies in its potential in relation to contamination source traceability. Accurate classification of different FPO types—whether polyethylene (PE), polypropylene (PP), polystyrene (PS), or other polymer variants—enables food processors to trace contamination back to specific sources within the production line. This traceability is essential for implementing targeted interventions to prevent contamination at its source, whether from packaging materials, processing machinery components, or other environmental origins [9,10]. By accurately identifying the specific FPO type, manufacturers can determine whether contamination originates from conveyor belts, packaging films, cleaning tools, or other plastic components in the processing environment, thereby enabling more effective quality control measures and equipment maintenance protocols.

Despite the importance of FPO detection and classification in food products, conventional imaging modalities remain insufficient for routine industrial applications. Traditional methods—including metal detection, X-ray imaging, and conventional color imaging—each have limitations when applied to the detection and classification of FPOs in meat products. Metal detection systems are fundamentally restricted to identifying metallic contaminants and are therefore ineffective against non-metallic FPOs. X-ray imaging, while useful for denser materials such as bone fragments, struggles to detect low-density plastics with densities close to that of chicken muscle tissue and cannot provide the chemical compositional information required to distinguish among FPO types. Similarly, conventional color imaging methods are often confounded by the visual similarity between FPOs and surrounding muscle or fatty tissues. Moreover, variability in the color of the same plastic type from different sources further increases the likelihood of false-positive and false-negative classification, thereby limiting the reliability of such approaches [11,12,13].

To overcome the shortcomings of conventional imaging, hyperspectral imaging (HSI) has emerged as a powerful, non-destructive technology that combines spectroscopy and imaging to capture both spectral and spatial information across a wide range of wavelengths. This enables detailed discrimination of different FM types, including various FPOs, based on their unique spectral signatures together with limited spatial features [14,15]. In the context of food safety, early HSI-based FPO detection and classification techniques relied primarily on chemometric workflows to identify polymer-specific spectral features. For instance, Faltynkova et al. reviewed the use of infrared-based HSI combined with multivariate analysis techniques such as principal component analysis (PCA), partial least-squares discriminant analysis (PLS-DA), and the successive projection algorithm (SPA) to classify dry plastic pieces, achieving accuracies of up to 95% under controlled laboratory conditions [16]. Similarly, Serranti et al. optimized a near-infrared (NIR) HSI-based system for the classification of PE, PP, and PS fragments on poultry meat, selecting five critical wavelengths via the SPA and employing PLS-DA to achieve classification accuracies ranging from 88% to 93% [17]. Another study demonstrated that combining PCA with support vector machine (SVM) classification enabled the effective identification of polyvinyl chloride (PVC), polyethylene terephthalate (PET), and low-density polyethylene (LDPE) fragments on pork surfaces, with accuracies exceeding 90% for larger fragments, although classification performance deteriorated substantially for smaller plastic pieces [18].

With the advancement of machine learning, deep learning (DL) approaches have increasingly outperformed traditional chemometric methods in hyperspectral FPO classification tasks because of their ability to automatically learn complex spatial–spectral features. Convolutional neural networks (CNNs) are the most widely adopted DL models for this purpose. For example, Sifnaios et al. developed a one-dimensional CNN architecture for pixel-level classification of four plastic types, while Maram et al. introduced a two-step DL pipeline that used YOLOv8 for initial detection and a one-dimensional CNN for subsequent classification of FPOs on chicken breast fillets [19,20]. These DL-based methods demonstrated improvements in detection and classification accuracy, robustness, and automation, thereby establishing new benchmarks for hyperspectral FPO analysis in food safety applications.

More recently, vision transformer-based models have emerged as powerful alternatives to CNNs for pixel-level hyperspectral image classification, demonstrating superior spatial–spectral feature extraction and improved performance in hyperspectral remote sensing applications [21,22]. Originally developed for natural language processing, these transformer architectures rely on self-attention to dynamically model correlations across spatial and spectral dimensions, thereby capturing complex long-range dependencies in hyperspectral data [23,24,25]. In contrast, CNNs, with their localized receptive fields and shared weights, often struggle to match the representational flexibility of transformer architectures [26,27,28]. Central to the success of transformers is the use of position encoding mechanisms, which explicitly incorporate positional information into input tokens—whether text, pixel, or patch tokens—so that the model can distinguish them by location rather than content alone. Because self-attention is permutation invariant and does not inherently preserve token order, positional information must be introduced explicitly. This substantially enhances the model’s ability to capture complex spatial–spectral patterns [23,29]. These capabilities have motivated the development of several task-specific spectral–spatial transformer models in the hyperspectral remote sensing domain, including HiT [22], LSGA [30], SpectralFormer [21], and the spectral–spatial feature token transformer (SSFTT) [31].

Despite their advantages, current vision transformer-based models face challenges when directly applied to HSI. The standard self-attention mechanism incurs quadratic computational complexity $O(n^{2})$ for a single hyperspectral patch with *n* pixels, leading to very high memory and computational requirements for the high-resolution hyperspectral datasets commonly encountered in real-world food processing lines [28,32]. Moreover, in typical transformer-based approaches, absolute positional embedding (APE) initially reduces the spatial arrangement of a 2D patch into a linear sequence, subsequently incorporating fixed embedding vectors derived from those linear indices. This often leads to a misrepresentation of genuine geometric relationships; for instance, vertically adjacent pixels might be given positions that imply a wider gap compared to horizontally adjacent ones, even when both are equidistant from the center pixel. The widespread application of APE in spatial–spectral transformers inadequately captures the essential relative spatial relationships needed for precisely modeling the complex spatial–spectral dependencies that are fundamental to hyperspectral imagery [33]. These limitations create a substantial gap between state-of-the-art transformer approaches and the practical demands of efficient and precise FPO classification in the poultry industry. To address these challenges, this study proposes a novel spatial–spectral transformer-based framework specifically optimized to classify 12 distinct FPO classes alongside a class representing normal skinless broiler breast meat, leveraging the strengths of transformer models in HSI. The main contributions of this work are summarized as follows:

1. We introduce a novel spatial–spectral transformer-based framework specifically designed for high-performance pixel-level classification of 12 distinct types of 5×5 mm FPOs on poultry meat using NIR-HSI. Our approach captures both the spectral and spatial context inherent in hyperspectral images through computationally efficient patch-based processing. It directly addresses the critical challenge of classifying small, transparent, and color-similar FPOs that conventional RGB-based systems often fail to identify reliably, thereby providing a practical approach for enhancing food safety in industrial poultry processing.
2. We propose a computationally efficient attention mechanism termed center-focused linear attention (CFLA). CFLA redesigns attention computation by using only the center pixel as the query while attending to all pixels within the local hyperspectral patch, thereby reducing computational complexity from $O(n^{2})$ to O(n) while preserving classification performance relative to conventional self-attention. This design enables the model to aggregate contextual information from surrounding pixels for center-pixel classification without incurring the quadratic scaling burden associated with patch size.
3. To overcome the limitations of APE in HSI-based vision transformers, we introduce a patch-local mixed-axis two-dimensional rotary position embedding (RoPE) mechanism within the transformer encoder block that preserves the inherent two-dimensional geometry of hyperspectral patches. Unlike conventional APE, which flattens spatial dimensions and weakens geometric relationships among pixels, the proposed position encoding scheme preserves the natural grid structure of the patch and effectively captures relative positional relationships, including diagonal, vertical, and horizontal dependencies.
4. To further improve model efficiency, we employ low-rank projection matrices for the query, key, value, and output transformations within the attention mechanism. This design reduces the number of trainable parameters and promotes a lightweight model architecture without compromising model capacity or classification performance.

## 2. Materials and Methods

### 2.1. Hyperspectral Dataset Collection and Preparation

The HSI system used in this study employs a pushbroom line-scanning configuration to acquire detailed spectral information in the 1000–1700 nm wavelength NIR range, as described in Section 2.1.3. This subsection describes the data collection and sample preparation procedures used to construct the hyperspectral dataset for FPO analysis.

#### 2.1.1. Foreign Plastic Objects Sample Preparation

**Figure 1:**
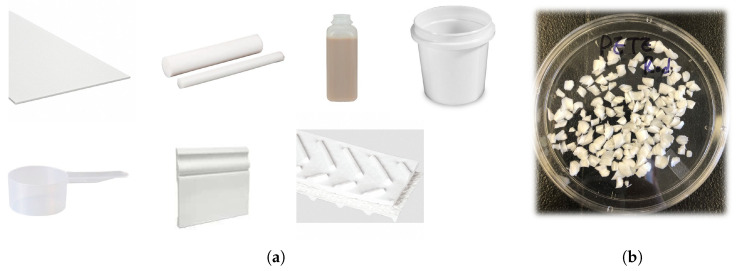
Various plastic material sources shown in (**a**), including polyethylene sheets, polypropylene rods, PET bottles, polystyrene containers, and fabric materials, were systematically cut into standardized 5×5 mm rectangular pieces to prepare FPO samples. The Petri dish in (**b**) contains representative PET FPOs prepared for hyperspectral image acquisition, detection, and classification on poultry meat.

The investigation focused on 12 polymer types commonly found in poultry processing facilities. These materials included the six main plastic types: PET, PP, PS, PVC, LDPE, and high-density polyethylene (HDPE). Additionally, specialized materials relevant to food processing operations were included, namely, polyurethane (PUR), which is commonly used in food-grade conveyor belts; acrylonitrile butadiene styrene (ABS); natural and synthetic rubber (RUB); fabric (FAB); nylon (NYL); and Teflon (TEF). Together, these materials represent a diverse set of polymeric contaminants that may enter the food processing stream through equipment wear, packaging materials, personal protective equipment, or maintenance activities. To capture within-class variability arising from different source materials, multiple material sources were collected for most polymer types before preparing the FPO samples. Specifically, six different material sources were used for each of PET, PP, PS, PVC, LDPE, and HDPE; three sources were used for each of PUR, RUB, FAB, NYL, and TEF; and one source was used for ABS. Each FPO sample was then prepared by cutting the original polymer sources into small rectangular pieces with nominal dimensions of 5×5 mm, as illustrated in Figure 1. This standardized size was selected to simulate realistic small contaminant dimensions in accordance with food safety regulatory guidance. Although the U.S. Department of Agriculture (USDA) does not define a universal size threshold for foreign plastic objects or other foreign materials in poultry, foreign materials in the 7–25 mm range are generally regarded as inherently hazardous, and even smaller materials may still pose risks, particularly for vulnerable populations such as infants and older adults.

#### 2.1.2. Sample Preparation

**Figure 2:**
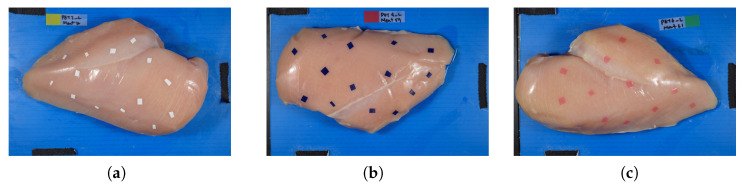
Representative color images of prepared chicken breast fillet samples containing 5 mm × 5 mm PET FPOs: (**a**) white; (**b**) dark blue; and (**c**) pink. These images illustrate representative sample preparation prior to hyperspectral imaging.

Fresh broiler breast fillets (n=52) were obtained from a local poultry processing plant and prepared as skinless, boneless chicken breast fillets. The fillets were selected to represent typical commercial poultry products while maintaining consistent tissue characteristics and surface properties. Each fillet was carefully positioned on the imaging platform with the skin side facing upward. The prepared FPO pieces were then placed randomly but uniformly on the surface of individual fillets to ensure representative coverage across different tissue regions, including muscle and fat deposits, as shown in Figure 2.

#### 2.1.3. HSI System Configuration

**Figure 3:**
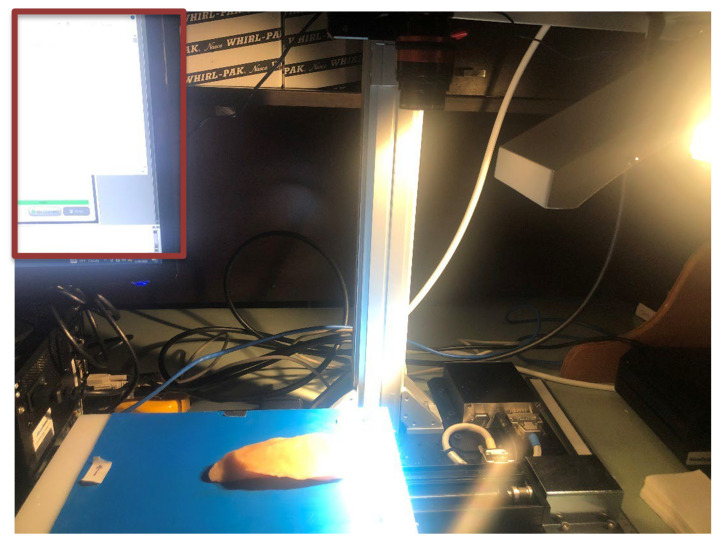
Pushbroom line-scanning hyperspectral imaging system (Micro-Hyperspec Extended VNIR, Headwall Photonics) operating at 600–1700 nm with an InGaAs detector that was reduced to 1000–1700 nm for the study. The system features a computer-controlled blue moving stage beneath the stationary sensor head and tungsten halogen illumination for uniform lighting during sample acquisition.

Hyperspectral data were acquired using a pushbroom line-scanning system (Micro-Hyperspec Extended VNIR R640, Headwall Photonics, Bolton, MA, USA) configured for extended visible and NIR spectral analysis. The system operates over a wavelength range of 600–1700 nm using an indium gallium arsenide (InGaAs) focal plane array detector coupled with a 25 mm objective lens. Although this broader spectral range could be used to capture both visible characteristics and NIR absorption features relevant to FPO classification and discrimination from surface biological tissues, this study used only the 1000–1700 nm range to align with the standard operating range of common industrial NIR HSI cameras. The imaging configuration employed a linear scanning approach in which samples were placed on a computer-controlled moving stage that translated beneath the stationary hyperspectral sensor head, as shown in Figure 3. Illumination was provided by tungsten halogen lamps positioned to ensure uniform lighting across the full scanning area. The scanning parameters were optimized to provide sufficient spatial resolution for detecting 5×5 mm FPOs while maintaining adequate spectral fidelity for material classification. The acquired raw hyperspectral images had dimensions of 638 (width) by 1068 (height, i.e., the number of line scans) by 268 spectral bands spanning 600–1700 nm. For all experiments in this study, only the 1000–1700 nm subset was retained, resulting in 171 spectral channels for analysis. The spectral sampling resolution was 4.1 nm, as determined by the average wavelength separation across the measured range.

#### 2.1.4. Image Calibration

Raw digital numbers (DNs) acquired by the hyperspectral imager were radiometrically calibrated to ensure accurate spectral measurements and to obtain relative spectral intensities while minimizing illumination-related variations. The calibration procedure involved acquiring both white-reference and dark-reference images. White-reference images were captured using a diffuse reflectance panel with 99% reflectivity (10” × 10” Spectralon, Labsphere, North Sutton, NH, USA), which provided a uniform diffuse reflectance standard across the full wavelength range. Dark-reference images were acquired by completely blocking the lens aperture with an opaque lens cap, thereby capturing the system’s inherent thermal and electronic noise. The calibration transformation was applied to each pixel of the raw hyperspectral image using the standard reflectance calibration equation:

$$\mathrm{Percent}\;\mathrm{Reflectance}\;\left(\%\right)=\frac{\mathrm{Measured}\;\mathrm{DN}-\mathrm{Dark}\;\mathrm{Reference}}{\mathrm{White}\;\mathrm{Reference}-\mathrm{Dark}\;\mathrm{Reference}}\times 100\%$$

This normalization procedure converts raw DNs to percentage reflectance values, enabling quantitative spectral analysis and comparison across different imaging sessions. The calibrated hyperspectral datasets therefore provided standardized reflectance spectra that could be directly compared with spectral libraries and further analyzed using chemometric methods.

#### 2.1.5. Region of Interest (ROI) Definition and Ground-Truth Establishment

Following calibration, each hyperspectral image was annotated by a human expert to establish precise ground-truth classifications for model training and performance evaluation. ROIs were manually delineated using ENVI (Environment for Visualizing Imagery) software version 4.8. The annotation process involved careful identification and segmentation of distinct material classes within each image, including background pixels corresponding to the blue conveyor belt surface, foreground biological tissues corresponding to lean meat and fat, and FPOs. This manual annotation procedure is critical for constructing accurate datasets for deep learning. The expert-defined ROIs ensured that spectral signatures were correctly attributed to their corresponding material classes, thereby providing the foundation for developing robust classification models capable of distinguishing among different FPOs as well as differentiating FPOs from natural biological components on poultry products. During ROI annotation, boundary pixels were excluded from the labeled target-pixel set when their spectra could contain mixed contributions from adjacent classes rather than representing a single material. This ensured that the supervised learning was based on hyperspectral pixels corresponding to a single class. Across the 52 calibrated hyperspectral images, the final annotated dataset contained 295,340 labeled target hyperspectral pixels from the 13 classes used for classification (12 FPO classes and 1 fillet class). Each target pixel contained spectral information across the 1000–1700 nm wavelength range (171 spectral bands) together with a corresponding ground-truth label. Background pixels were annotated during ROI definition but were not included in the 13-class target-pixel classification set. For model development, the data split was performed at the labeled target-pixel level. To avoid class imbalance during training and validation, class-balanced random sampling was used to select 2400 target pixels per class for training (31,200 total) and 1200 target pixels per class for validation (15,600 total). All remaining labeled target pixels were assigned to the test set, resulting in 248,540 test pixels. During the patchification stage, one 7×7×171 hyperspectral cube was extracted for each target pixel; therefore, these counts correspond directly to 31,200 training cubes, 15,600 validation cubes, and 248,540 test cubes. Overall, this corresponds approximately to 10.6%, 5.3%, and 84.1% of the labeled target pixels for training, validation, and testing, respectively.

### 2.2. Proposed Transformer-Based Framework for Pixel-Level Classification

#### 2.2.1. Overview

**Figure 4:**
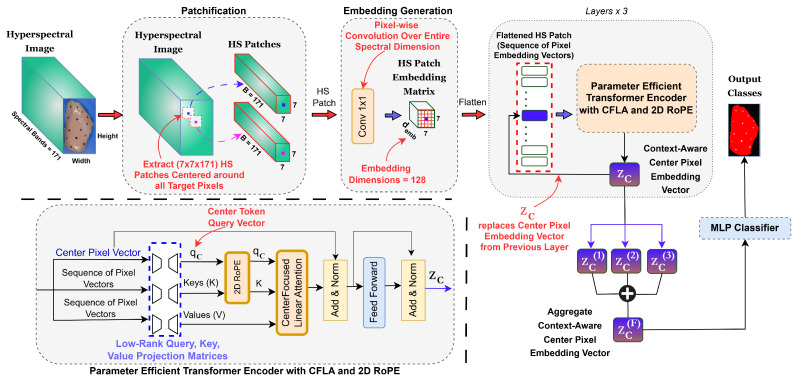
Proposed spatial–spectral transformer framework for pixel-level classification of FPOs on poultry meat using NIR HSI. The framework consists of four main stages: (1) patchification, in which 7×7×171 hyperspectral patches centered on each target pixel are extracted from the hyperspectral image; (2) embedding generation, in which a pixel-wise 1×1 convolution across the full spectral dimension transforms each pixel’s spectral signature into a $d_{\mathrm{emb}}$-dimensional embedding while preserving the spatial structure; (3) parameter-efficient transformer encoding, in which the sequence of pixel embeddings is processed through three consecutive encoder layers, each incorporating CFLA, low-rank projections, and patch-local mixed-axis two-dimensional RoPE; and (4) MLP classification, in which the context-aware center-pixel embeddings from all encoder layers are aggregated through a weighted sum to produce the final pixel-level prediction for the 13 classes (12 FPO types and 1 normal meat tissue class).

The complete workflow of our proposed framework, illustrated in Figure 4, implements a novel spatial–spectral processing pipeline specifically designed for accurate and efficient pixel-level classification of FPOs on poultry meat using NIR HSI. The framework comprises four interconnected stages: **(1) Patchification**: for each target pixel in the hyperspectral image, we extract a 7×7 patch centered on that pixel, creating a local context window for each target pixel. Each pixel in the 7×7 patch is represented by a 171-dimensional spectral vector. **(2) Embedding generation**: a pixel-wise 1 × 1 convolution (one-dimensional convolution) is applied across the entire spectral dimension of each pixel within the patch, transforming the spectral signature into $d_{emb}$-dimensional embedding vectors. This operation produces an embedding tensor of size $R^{d_{emb}\times 7\times 7}$ that preserves the spatial layout while capturing discriminative spectral features. The convolution operates independently on each pixel, thereby retaining the full spectral information required for material identification. **(3) Parameter-efficient transformer encoder with CFLA and 2D RoPE**: the embedding tensor is flattened into a sequence of 49 hyperspectral pixel embedding vectors and processed through the three consecutive transformer encoder layers. The output context-aware center-pixel embedding vector from each layer replaces the center-pixel embedding in the sequence passed to the next encoder layer. Each encoder layer incorporates three key innovations: (a) low-rank factorized projections (LRP) for query, key, value, and output matrices to reduce parameters; (b) CFLA, which computes attention using only the center-pixel query vector against all pixels in the patch as keys, achieving O(n) computational complexity; and (c) patch-local mixed-axis 2D RoPE with learnable frequencies to encode relative spatial positions while preserving the two-dimensional grid geometry. The resulting context-aware center-pixel embeddings from all three encoder layers are retained in a list for later aggregation. **(4) Multi-layer perceptron (MLP) classifier**: the framework then aggregates the context-rich center-pixel embeddings from all layers through a learnable weighted sum, capturing multi-scale spatial–spectral features. This aggregated representation is then processed through a two-layer MLP classifier to produce the final classification logits for the 13 classes (12 distinct FPO classes and 1 muscle/fat class).

#### 2.2.2. Patchification

Given a hyperspectral image of size Height×Width×SpectralBands, we assume that the entire hyperspectral image consists of *N* target hyperspectral pixels, where each target hyperspectral pixel belongs to one of the 13 classes. In our dataset, all the hyperspectral images had a height of 1068, a width of 638, and 171 spectral bands, whereas the number of target hyperspectral pixels, *N*, varied across the 52 images. To effectively leverage both the spectral and spatial information inherent in HSI data, the proposed framework first extracts a square local patch centered on each target hyperspectral pixel, referred to as the *center pixel*, with spatial dimensions of 7×7 representing the height and width of the patch. This extraction step provides the immediate local neighborhood context required for subsequent processing. As a result, the total number of extracted patches is equal to the number of target pixels to be classified. Each extracted patch is then passed to the embedding generation module.

#### 2.2.3. Embedding Generation

In contrast to conventional 2D spatial convolutions that blend spatial and channel information simultaneously, we employ 2D convolution with 1×1 convolutional filters (point-wise convolutions), focusing specifically on spectral dimension interactions at each spatial location within the patch. This operation reduces spectral dimensionality and promotes cross-channel interactions while preserving spatial dimensions. Such a convolution layer efficiently captures complex, nonlinear channel-wise features with minimal spatial interference, which is particularly beneficial for HSI, where spectral variability often carries greater discriminative significance than spatial textural details during initial feature extraction.

In our approach, given an input hyperspectral patch denoted by $P\in R^{B\times Patch_{H}\times Patch_{W}}$, where B=171 spectral bands and $Patch_{H}$, $Patch_{W}$ are both equal to 7, we generated local embeddings for each pixel by applying a two-dimensional convolution with a 1×1 filter across the full spectral dimension:

(1) $$E=\mathrm{ReLU}\left(\mathrm{BN}(W*P)\right),$$

where $W\in R^{d_{\mathrm{emb}}\times B\times 1\times 1}$ denotes the convolution kernel that projects the original spectral vectors into the embedding space, $d_{\mathrm{emb}}=128$ is the embedding dimension, BN denotes batch normalization, ReLU denotes the rectified linear unit activation, and E denotes the resulting patch embedding tensor. Thus, $E\in R^{d_{\mathrm{emb}}\times 7\times 7}$, which is subsequently flattened into a sequence X of 49 pixel embedding vectors.

By adopting 1×1 filters as our spectral feature extractors, we harness their ability to perform channel-wise pooling and dimensionality reduction with minimal overhead. This design choice ensures that each pixel’s embedding encapsulates its full spectral profile, setting the stage for subsequent modules—such as transformer encoder blocks—to effectively capture spatial and contextual relationships. Ultimately, this strategy leverages the proven advantages of point-wise convolutions in CNNs while adapting these insights to the unique requirements of HSI contexts [34,35,36].

#### 2.2.4. Parameter-Efficient Transformer Encoder with CFLA and 2D RoPE

The proposed efficient transformer encoder integrates three main components: (a) parameter-efficient LRPs; (b) the proposed computationally efficient CFLA mechanism; and (c) patch-local mixed-axis 2D RoPE with learnable frequencies as the position encoding mechanism.

##### Parameter-Efficient Low-Rank Projections

In conventional transformer encoders, each projection matrix for queries, keys, values, and outputs typically has dimensions $d_{\mathrm{emb}}\times d_{\mathrm{emb}}$, resulting in $d_{\mathrm{emb}}^{\;2}$ parameters per matrix and, consequently, a large overall parameter count. To reduce the number of trainable parameters while maintaining representational capacity, we decompose each full-rank projection matrix $W\in R^{d_{\mathrm{emb}}\times d_{\mathrm{emb}}}$ into a lower-rank approximation as follows:

(2) $$W=W_{2}W_{1}$$

where $W_{1}\in R^{d_{\mathrm{emb}}\times d_{\mathrm{bottleneck}}}$ and $W_{2}\in R^{d_{\mathrm{bottleneck}}\times d_{\mathrm{emb}}}$, with $d_{\mathrm{bottleneck}}=32$. This factorization reduces the original $d_{\mathrm{emb}}^{\;2}$ parameters to $2\times d_{\mathrm{emb}}\times d_{\mathrm{bottleneck}}$, corresponding to an approximate 50% reduction in the conventional query, key, value, and output projection layers, i.e., $W_{q}$, $W_{k}$, $W_{v}$, and $W_{\mathrm{out}}$, when $d_{\mathrm{bottleneck}}=d_{\mathrm{emb}}/4$. We apply this strategy uniformly across all projection layers to achieve substantial parameter reduction while preserving expressive capacity.

##### Center-Focused Linear Attention

**Figure 5:**
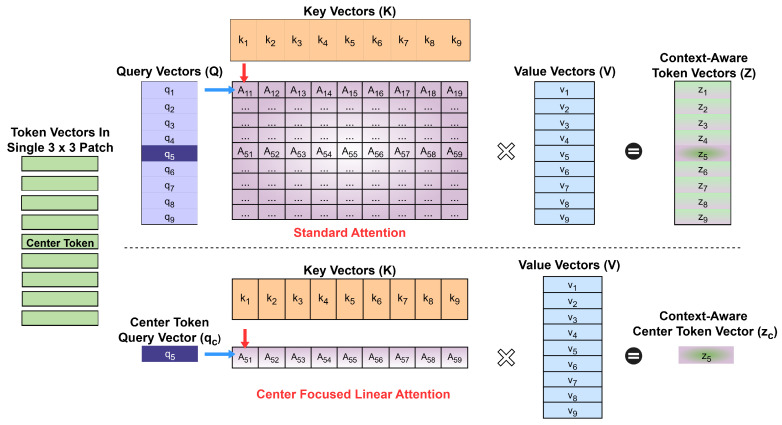
Schematic comparison of computational patterns in standard self-attention (**top**) and the proposed CFLA (**bottom**) using a 3×3 patch for illustration. Standard self-attention computes all query–key interactions, resulting in $O(n^{2})$ complexity, whereas CFLA uses only the center token as the query against all keys, reducing the complexity to O(n) while preserving contextual information for center-pixel classification.

Standard self-attention computes queries, keys, and values for all tokens. In contrast, the proposed CFLA mechanism focuses on the *center token*, denoted by $x_{c}$, corresponding to the center pixel in the flattened sequence *X*. For a sequence of length *n*, the center token index is defined as c=⌊n/2⌋. CFLA computes attention only for the center token while using all tokens in the sequence as keys and values. Figure 5 illustrates the computational difference between CFLA and conventional self-attention for a single patch when flattened. Formally,

(3) $$\begin{array}{cc} q_{c} & =W_{q}^{2}W_{q}^{1}x_{c},\;K=W_{k}^{2}W_{k}^{1}X,\;V=W_{v}^{2}W_{v}^{1}X, \end{array}$$

where $q_{c}$ denotes the query vector of the center token, and K and V denote the keys and values for all tokens in the sequence of pixel embeddings. Before computing attention scores, mixed-axis 2D RoPE is applied to the query and keys to encode positional information:

(4) $$\begin{array}{cc} q_{c}^{\prime } & ={\mathrm{RoPE}}_{\mathrm{mixed}}\left(q_{c}\right),\;K^{\prime }={\mathrm{RoPE}}_{\mathrm{mixed}}\left(K\right), \end{array}$$

where ${\mathrm{RoPE}}_{\mathrm{mixed}}$ is described in the following subsection. The attention scores are then computed using only the center token transformed query and all transformed keys:

(5) $$\begin{array}{cc} A & =\mathrm{softmax}\left(\frac{q_{c}^{\prime }{K^{\prime }}^{T}}{\sqrt{D_{\mathrm{head}}}}\right),\;o=AV, \end{array}$$

where $D_{\mathrm{head}}$ denotes the dimensionality of each attention head. Multi-head self-attention extends this formulation by performing *k* attention operations, referred to as *heads*, in parallel and concatenating their outputs:

(6) $$o_{c}=\mathrm{Concat}\left(o_{i},\ldots ,o_{k}\right),$$

(7) $$o_{c}^{\prime }=W_{\mathrm{out}}^{2}W_{\mathrm{out}}^{1}o_{c},$$

where $o_{i}$ denotes the output of the *i*th attention head, and $W_{\mathrm{out}}^{1}$ and $W_{\mathrm{out}}^{2}$ are the LRP head, that is, the factorized output projection matrices.

The complete architecture contains L=3 encoder layers, each composed of the center-focused attention mechanism and feed-forward components. Each encoder layer produces a context-aware center-pixel representation denoted by $z_{c}^{\left(l\right)}$, where *l* indicates the encoder layer index. The representation produced by each layer replaces the center-pixel embedding in the input sequence to the next layer. In addition, the center-pixel representations from all three encoder layers are retained in a list for later aggregation. The final aggregated center-pixel representation is computed as follows:

(8) $$\begin{array}{c} z_{c}^{F}=\sum_{l=1}^{3}w^{\left(l\right)}z_{c}^{\left(l\right)}, \end{array}$$

where $w^{\left(l\right)}$ denotes the learnable aggregation weight for the *l*th encoder layer. This aggregated context-rich representation of the center pixel is then passed to the MLP classifier.

##### Patch-Local Mixed-Axis 2D RoPE with Learnable Frequencies

To capture diagonal and off-axis spatial relationships within each hyperspectral patch, we employ a mixed-axis 2D RoPE that combines horizontal (Δx) and vertical (Δy) offsets into a single complex rotation before attention is computed:

Reqckn∗eiθx(cx−nx)+θy(cy−ny)

where $\left(c_{x},c_{y}\right)$ and $\left(n_{x},n_{y}\right)$ denote the coordinates of the center token and an arbitrary token in the patch, respectively, and $\theta^{x}$ and $\theta^{y}$ are learnable frequency parameters. This formulation allows the attention mechanism to flexibly combine horizontal and vertical displacements, thereby capturing diagonal and off-axis spatial relationships that commonly arise in hyperspectral imagery. By updating $\theta^{x}$ and $\theta^{y}$ during training, the model adapts to different spatial scales and orientations, improving its ability to represent complex spatial–spectral patterns. The full mathematical formulation, including the initialization of $\theta^{x}$ and $\theta^{y}$ and their application across the P×P patch grid, is provided in Appendix A.

#### 2.2.5. MLP Classifier

The final aggregated center-pixel representation, $z_{c}^{F}$, obtained after the three transformer encoder layers, is passed through a two-layer MLP with batch normalization, dropout, and ReLU activation, followed by a linear classifier to produce the class logits:

(9) $$\begin{array}{c} y=\mathrm{Linear}\left(\mathrm{ReLU}(\mathrm{BN}\left(\mathrm{Dropout}\left(\mathrm{Linear}\left(z_{c}^{F}\right)\right)\right))\right) \end{array}$$

where $y\in R^{K}$ and *K* denotes the number of output classes.

### 2.3. Training Setup and Computing Environment

Training was performed for 200 epochs using an early-stopping patience of 100 epochs, an initial learning rate of 0.001, and a batch size of 80. All experiments were conducted on a Lenovo IdeaCenter 5i workstation (Lenovo Group Limited, Morrisville, NC, USA) equipped with a 12^th^-generation Intel^®^ Core^TM^ i7 CPU (2.10 GHz) (Intel, Santa Clara, CA, USA) and an NVIDIA GeForce RTX 3060 GPU (Nvidia, Santa Clara, CA, USA) with 12 GB of VRAM and 3584 CUDA cores. All implementations were developed in PyTorch version 3.11 and executed with CUDA version 12.1.1 to accelerate GPU computation.

### 2.4. Evaluation Metrics

#### 2.4.1. Performance Metrics

To evaluate pixel-level classification performance, we report overall accuracy (OA), average accuracy (AA), Cohen’s Kappa coefficient (κ), class-wise recall, class-wise precision, and class-wise F1-score. OA measures the proportion of correctly classified pixels over the entire test set, whereas AA measures the mean recall across all classes. The Kappa coefficient quantifies agreement between predicted and reference labels while accounting for agreement expected by chance. In addition, class-wise recall, precision, and F1-score provide a more detailed assessment of performance for individual classes. These metrics are defined as follows:

$$OA=\frac{\sum_{i=1}^{N_{\mathrm{classes}}}TP_{i}}{N_{\mathrm{test}}}$$

$$AA=\frac{\sum_{i=1}^{N_{\mathrm{classes}}}R_{i}}{N_{\mathrm{classes}}}$$

$$\kappa =\frac{p_{o}-p_{e}}{1-p_{e}}$$

$${\mathrm{Recall}}_{i}=\frac{TP_{i}}{TP_{i}+FN_{i}}$$

$${\mathrm{Precision}}_{i}=\frac{TP_{i}}{TP_{i}+FP_{i}}$$

$$F1_{i}=\frac{2\;{\mathrm{Precision}}_{i}\;{\mathrm{Recall}}_{i}}{{\mathrm{Precision}}_{i}+{\mathrm{Recall}}_{i}}$$

where $TP_{i}$, $FP_{i}$, and $FN_{i}$ denote the true positives, false positives, and false negatives for class *i*, respectively; $N_{\mathrm{test}}$ is the total number of pixels in the test set; and $N_{\mathrm{classes}}$ is the total number of classes. Here, ${\mathrm{Recall}}_{i}$, ${\mathrm{Precision}}_{i}$, and $F1_{i}$ denote the recall, precision, and F1-score for class *i*, respectively. The terms $p_{o}$ and $p_{e}$ denote the observed and expected agreement derived from the confusion matrix (CM). Specifically, $p_{o}$ is obtained by dividing the sum of the diagonal entries of the CM by the total number of samples, whereas $p_{e}$ is computed as $\frac{\sum_{i=1}^{N}\left(A_{i}\times B_{i}\right)}{N^{2}}$, where $A_{i}$ and $B_{i}$ are the row-wise and column-wise sums of the CM, respectively, and *N* is the total number of samples. Together, these metrics provide a comprehensive assessment of the model’s reliability and discriminative performance for FPO classification. In addition to the quantitative evaluation, classification maps are also visualized to support qualitative comparison of the predicted results.

#### 2.4.2. Efficiency Metrics

To evaluate the efficiency of the proposed framework, we report both architecture-level and empirical inference metrics. The architecture-level metrics include multiply–accumulate operations (MACs), floating-point operations (FLOPs), the number of trainable parameters, and model size. In addition, empirical inference efficiency is evaluated using latency per sample and throughput. Here, one sample corresponds to a single hyperspectral patch cube of size 7×7×171 centered on a target pixel.

##### Computational Complexity

Computational complexity is quantified using MACs and FLOPs. MACs measure the total number of multiply–accumulate operations required during a forward pass, whereas FLOPs represent the total number of floating-point arithmetic operations. These metrics are used to characterize the computational cost of the model architecture.

##### Model Parameters

The number of trainable parameters is obtained by summing all learnable weights across the embedding generation layer, projection matrices, feed-forward sublayers, normalization layers, and the MLP classifier. This provides an exact count of the trainable parameters in the full model for memory efficiency.

##### Model Size

Model size is reported as the size of the serialized parameter file in megabytes (MB) under single-precision storage. This metric reflects the memory footprint of the deployable model as stored on disk or loaded into GPU memory.

##### Inference Latency and Throughput

To assess practical inference efficiency, we report latency per hyperspectral patch cube (ms) and throughput in terms of hyperspectral patch cubes processed per second (patches/s). Latency per patch measures the average inference time required to process one hyperspectral patch cube, whereas throughput measures the number of hyperspectral patch cubes processed per second. In the ablation experiments, these metrics were measured at a batch size of 1024 over 1000 timed runs after 50 warmup iterations. Speedup was computed relative to the baseline configuration without LRP and without CFLA.

## 3. Results and Discussion

This section presents a comprehensive evaluation of the proposed transformer-based framework for pixel-level classification of plastic contaminants in NIR hyperspectral images. We first report the overall quantitative and qualitative classification performance of the proposed model, followed by an efficiency analysis, positional encoding comparisons, a hyperparameter sensitivity analysis, and the discussion.

### 3.1. Overall Classification Performance

#### 3.1.1. Quantitative Results

To quantitatively assess the performance of the proposed framework, we compared it with representative CNN- and transformer-based HSI classification models, including 2D-CNN, 3D-CNN, SpectralFormer, HiT, and LSGA, as summarized in Table 1. Because benchmark spatial–spectral models specifically developed for pixel-level FPO classification in food inspection remain limited, these baselines were selected from the hyperspectral remote sensing literature, where pixel-wise HSI classification is also a core task. The proposed model achieved the strongest overall performance among all compared methods, with an OA of 99.39 ± 0.11%, an AA of 99.57 ± 0.15%, and a Cohen’s Kappa coefficient of 99.31 ± 0.18. Among the baseline models, LSGA achieved the closest performance, with an OA of 96.36 ± 0.82%, an AA of 97.09 ± 0.64%, and a Kappa of 95.30 ± 0.86, followed by SpectralFormer with an OA of 94.94 ± 0.69% and HiT with an OA of 90.04 ± 0.64%. The CNN-based baselines performed substantially worse, with OA values of 79.61 ± 0.67% for 2D-CNN and 86.81 ± 0.61% for 3D-CNN. These results indicate that the proposed framework provides clear performance gains over both conventional CNN-based approaches and recent spatial–spectral transformer architectures under the same evaluation setting. At the class level, the proposed model achieved the highest recall for most categories, including Fillet, ABS, FAB, HDPE, PET, PP, PS, PUR, PVC, RUB, and TEF. For LDPE and NYL, the proposed method remained highly competitive, with recall values of 99.19 ± 0.19% and 99.64 ± 0.15%, respectively, although 3D-CNN achieved slightly higher recall for these two classes. Particularly notable improvements were observed for challenging classes such as PET, PP, and PS, where the CNN baselines and several transformer baselines exhibited substantially lower recall. The consistently strong performance across both the fillet class and the diverse polymer categories indicates that the proposed framework can reliably discriminate between biological tissue and multiple visually or spectrally similar FPO types. To further characterize the predictive behavior of the proposed model, Table 2 reports per-class precision, recall, and F1-score. The model maintained uniformly high precision across all classes, ranging from 98.05 ± 0.16% for NYL to 99.86 ± 0.18% for Fillet, while recall ranged from 98.59 ± 0.15% for Fillet to 99.98 ± 0.19% for TEF. The resulting F1-scores were similarly stable, ranging from 98.76 ± 0.12% for PP and 98.84 ± 0.11% for NYL to 99.84 ± 0.13% for FAB and 99.84 ± 0.11% for PVC. Common packaging-related polymers such as PET, PP, HDPE, and LDPE, as well as industrially relevant materials such as PUR, PVC, RUB, and TEF, were all classified with very high precision and recall. Overall, these results show that the proposed framework not only achieves the best overall performance among the compared methods but also maintains highly reliable and balanced class-wise behavior across all evaluated material categories.

#### 3.1.2. Qualitative Results

**Figure 6:**
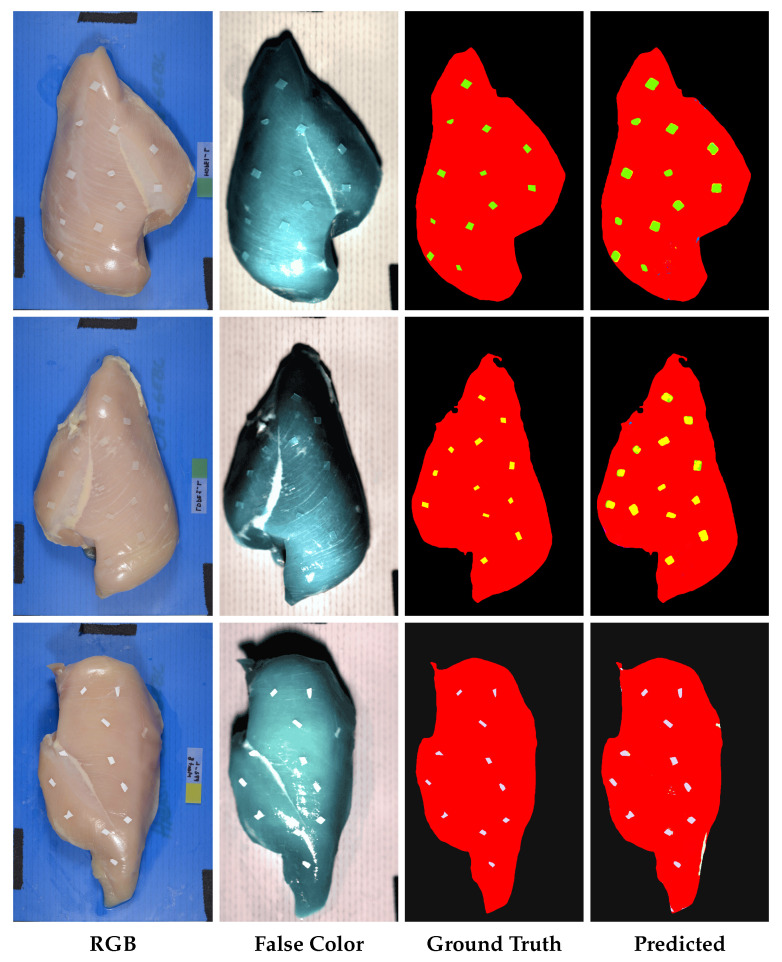
Representative qualitative classification results for three polymer types. From top to bottom, the **rows** correspond to HDPE, LDPE, and PP, respectively. From left to right, the **columns** show the RGB image, false-color composite, ground-truth annotation, and predicted classification map. The predicted maps closely match the ground truth, demonstrating spatially coherent and accurate pixel-level FPO classification.

The qualitative results further demonstrate the spatial accuracy and visual coherence of the proposed framework. Figure 6 presents representative examples for three polymer types: HDPE, LDPE, and PP. Each row shows the complete analysis sequence from the RGB image and false-color visualization to the ground-truth annotation and predicted classification map. The RGB images highlight the potential limitations of conventional color-based inspection, as some polymers exhibit visual appearances similar to those of biological tissues, particularly in regions with varying fat content. In contrast, the false-color composites reveal the enhanced discriminative capability of HSI. The predicted classification maps show that the proposed framework consistently identifies and localizes the FPOs with high spatial precision, producing clean boundaries and minimal classification noise. The close correspondence between the ground-truth and predicted maps indicates that the model can accurately delineate contaminant regions while avoiding excessive false-positive predictions. The consistent qualitative performance across different polymer types further supports the robustness of the proposed approach. These visual results are well aligned with the quantitative results reported in Section 3.1.1, confirming that the strong numerical performance of the proposed framework translates into spatially coherent and practically meaningful classification outcomes. Taken together, the qualitative and quantitative results indicate that the proposed method provides reliable pixel-level FPO classification suitable for food safety inspection applications.

### 3.2. Effectiveness of Model Components and Efficiency Analysis

To assess the contribution of the proposed architectural components, we conducted an ablation study focusing on LRP and center-focused linear attention (CFLA), as summarized in Table 3. The classification metrics are reported as mean ± standard deviation over 10 runs with different predefined random seeds, whereas the architecture-level efficiency metrics are deterministic quantities and are therefore reported once. The baseline configuration without LRP and CFLA exhibited the highest computational complexity, with 22.51 M MACs and 45.03 M FLOPs, as well as the largest parameter count and model size (451.2 K parameters and 1.72 MB), while achieving an OA of 99.37 ± 0.18%, an AA of 99.56 ± 0.16%, and a Kappa of 99.29 ± 0.21. Incorporating CFLA alone reduced MACs and FLOPs to 6.55 M and 13.11 M, respectively, while maintaining comparable classification performance. The configuration combining both LRP and CFLA modules achieved the most favorable overall trade-off, yielding the lowest computational cost (3.80 M MACs and 7.60 M FLOPs), the smallest model size (1.35 MB), and a mean classification performance that was the highest among the three configurations by a small margin, with an OA of 99.39 ± 0.11%, an AA of 99.57 ± 0.15%, and a Kappa of 99.31 ± 0.18. These results show that the proposed architectural components substantially improve computational and parameter efficiency while preserving strong and stable predictive performance.

To determine whether the architecture-level efficiency gains also translate into practical inference benefits, we further evaluated empirical inference efficiency at a default inference batch size of 1024, as reported in Table 4. The baseline model without LRP and CFLA achieved a latency of 0.0197 ± 0.0004 ms per hyperspectral patch cube and a throughput of 50,885.5 ± 919 patch cubes/s. Adding CFLA alone reduced the latency to 0.0052 ± 0.0003 ms and increased throughput to 192,310.0 ± 938 patch cubes/s, corresponding to a 3.78× speedup. The final configuration with both LRP and CFLA provided the best empirical inference efficiency, achieving a latency of 0.0047 ± 0.0000 ms per patch cube and a throughput of 212,971.5 ± 834 patch cubes/s, corresponding to a 4.19× speedup relative to the baseline. Together, Table 3 and Table 4 show that LRP and CFLA improve not only architecture-level efficiency but also practical inference performance.

**Figure 7:**
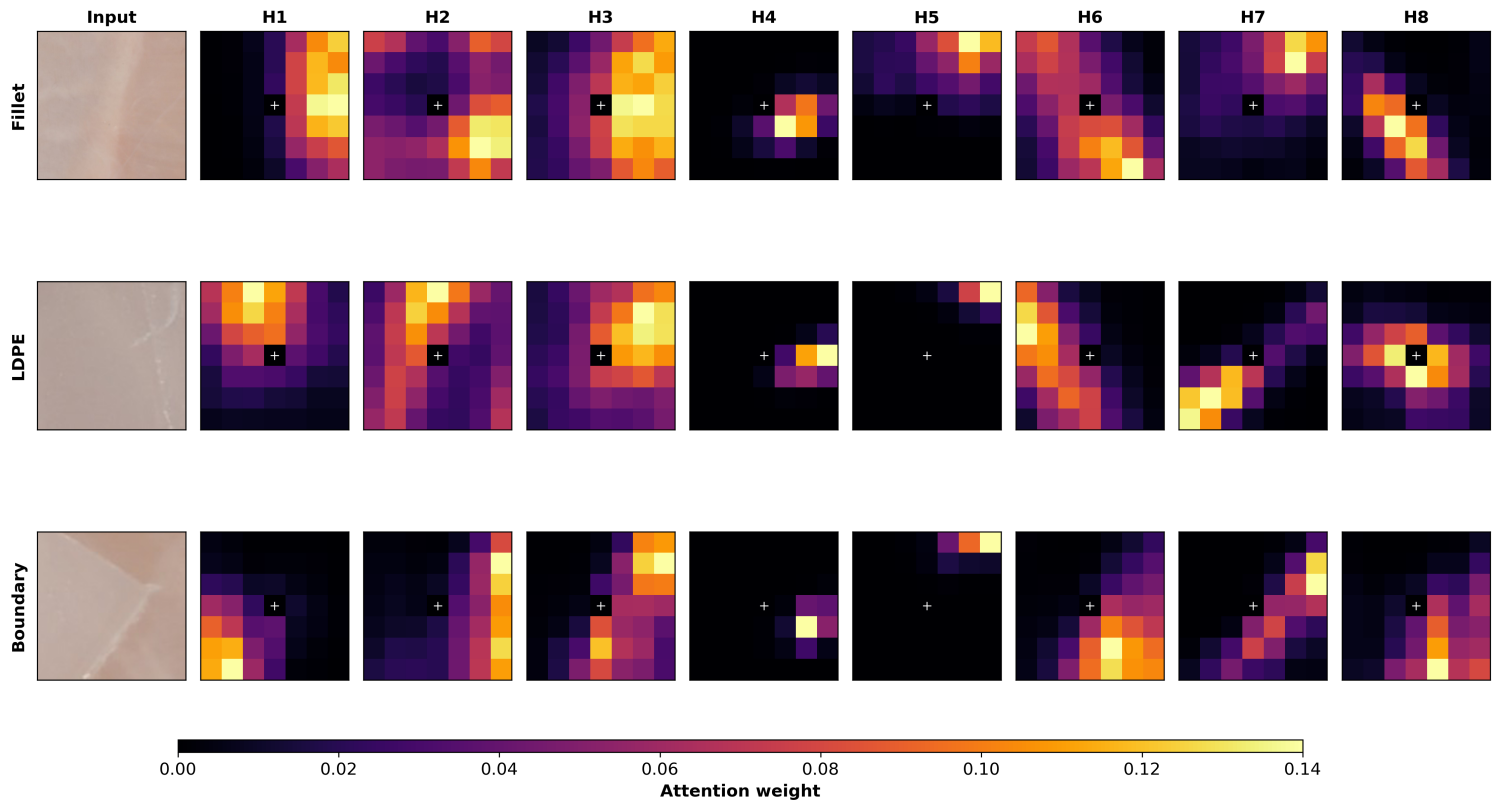
Representative CFLA attention weight distributions for three 7×7 patches from different local spatial contexts: a homogeneous fillet region (**top**), a homogeneous LDPE region (**middle**), and a near-boundary region between plastic and fillet (**bottom**). The first column shows the corresponding input patches, and columns H1–H8 show the attention maps for the eight heads in the second encoder layer. In each case, the center pixel (white cross) serves as the query. The heatmaps show that CFLA assigns non-uniform weights to surrounding pixels and adapts its attention pattern according to the local spatial–spectral context.

To further examine the behavior of CFLA, we visualized the attention weight distributions for representative 7×7 patches from three local spatial contexts, as shown in Figure 7. Specifically, the three inputs correspond to a homogeneous fillet patch, a homogeneous LDPE patch, and a near-boundary patch containing both LDPE and fillet pixels. In each case, the center pixel, indicated by the white cross, serves as the single query, and the resulting attention weights over all patch locations are displayed as 7×7 heatmaps for the eight attention heads in the second encoder layer. The attention maps show that CFLA assigns non-uniform weights across the local neighborhood, with attention distributions varying across heads and patch types. These qualitative observations are consistent with the intended role of CFLA in aggregating surrounding spatial–spectral information to refine the representation of the center pixel.

### 3.3. Comparison of Position Embedding Mechanisms

To evaluate the benefit of the patch-local mixed-axis 2D RoPE with learnable frequencies, we compared it with absolute positional embedding (APE), as summarized in Table 5. The 2D RoPE achieved the best overall performance, with an OA of 99.39 ± 0.11%, an AA of 99.57 ± 0.15%, and a Kappa of 99.31 ± 0.18. In contrast, APE achieved an OA of 99.03 ± 0.18%, an AA of 99.18 ± 0.22%, and a Kappa of 98.96 ± 0.16. These results indicate that preserving the two-dimensional patch geometry while encoding relative positional relationships improves the discrimination of spatial–spectral patterns in hyperspectral patches.

### 3.4. Impact of Architectural Hyperparameters

**Figure 8:**
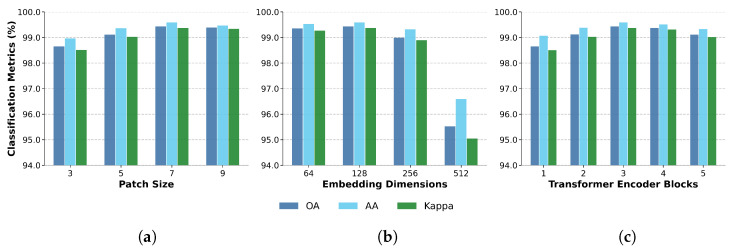
Sensitivity analysis of classification performance with respect to (**a**) patch size, (**b**) embedding dimension, and (**c**) the number of transformer encoder layers. Performance is reported in terms of OA, AA, and Kappa.

Figure 8 shows the effect of three key architectural hyperparameters—patch size, embedding dimension, and the number of transformer encoder layers—on classification performance. Increasing the patch size from 3 to 7 leads to clear improvements in OA, AA, and κ, whereas further enlargement yields only marginal gains. A similar trend is observed for the embedding dimension: increasing it from 64 to 128 improves all three metrics, but further increases to 256 and 512 lead to substantial performance degradation, likely because of over-parameterization and overfitting. The best overall configuration is achieved with three transformer encoder layers, beyond which no further performance gains are observed. These results support the architectural choices described in Section 2.2 and highlight the importance of balancing model capacity and generalization in pixel-level FPO classification.

### 3.5. Discussion

The results presented in this study demonstrate a substantial advancement in automated FPO classification for food safety applications through three primary technical contributions. The CFLA mechanism represents a fundamental shift in attention computation for hyperspectral imagery, where restricting attention to center-pixel queries dramatically reduces computational complexity while preserving essential spatial–spectral relationships. This design choice aligns with the practical reality that, in pixel-level classification tasks, the primary objective is to accurately classify the center pixel using contextual information from its neighborhood. The linear scaling with patch size O(n) versus $O(n^{2})$ addresses a critical bottleneck in transformer-based approaches for high-resolution hyperspectral data commonly encountered in industrial processing lines. In addition, the inference efficiency analysis showed that the final configuration with both CFLA and LRP achieved a 4.19× speedup relative to the baseline model, indicating that the efficiency gains are not only theoretical but also translate into practical inference-time benefits. Additionally, the introduction of patch-local mixed-axis 2D RoPE with learnable frequencies addresses a significant limitation in existing spatial–spectral transformers from remote sensing. Traditional APE flattens 2D patch structures into linear sequences, fundamentally misrepresenting geometric relationships within patches that are important for hyperspectral analysis. Our approach preserves the inherent grid structure while enabling the model to learn optimal frequency parameters for capturing both axis-aligned and diagonal spatial dependencies. This is particularly relevant for FPO identification, where contaminant boundaries often exhibit irregular shapes and orientations that require flexible spatial modeling capabilities. Moreover, the low-rank factorization strategy provides an effective solution for improving parameter efficiency within the transformer architecture without compromising representational capacity. The approximate 50% reduction achieved in the projection layers demonstrates that full-rank projections in transformer architectures contain substantial redundancy. This finding has broader implications for deploying DL models in resource-constrained and high-throughput industrial environments where memory and computational limitations are critical factors.

Secondly, the ablation studies provide valuable insights into the architectural design choices. The optimal configuration of 7×7 patches, 128-dimensional embeddings, 32-rank projection matrices, and three transformer encoder layers represents a careful balance between model capacity and generalization ability. The performance degradation observed with larger embedding dimensions (256 and 512) suggests that excessive parameterization leads to overfitting on the spectral signatures, highlighting the importance of architectural parsimony in hyperspectral analysis applications.

From a food safety perspective, our framework’s ability to accurately classify different polymer types has important implications for contamination source traceability. The high precision in distinguishing between packaging-derived plastics (PET, PP, and PS) and equipment-derived materials (PUR from conveyor belts and TEF from processing surfaces) enables food processors to implement targeted preventive measures. This capability transforms FPO identification from a reactive quality control measure into a proactive contamination prevention tool, potentially reducing product recalls and associated economic losses. The consistent performance across diverse polymer types, including those with similar spectral signatures, further validates the robustness of our spatial–spectral feature extraction approach. The framework’s success in classifying industrial materials such as PUR (99.81% F1-score) and TEF (99.83% F1-score) is particularly relevant for food processing environments where these materials are commonly present. Even for challenging materials such as NYL, which showed the lowest performance metrics, the model maintained precision above 98%, indicating reliable discriminative capability across all evaluated contaminant types.

### 3.6. Limitations

Despite the promising results, several limitations warrant consideration in future research and practical implementation: First, our evaluation focused on 5×5 mm FPO samples, which represent the lower end of the practical FPO size range and overlap with the larger end of the microplastic size spectrum. Real-world contamination scenarios may involve smaller fragments up to 2 mm, and the model’s performance on fragments smaller than 5 mm remains unexplored. The spatial resolution limitations of current HSI systems may therefore require alternative approaches or hardware improvements for reliable identification of smaller contaminants. Second, although the dataset included 12 common polymer types, the diversity of plastics and other materials encountered in industrial environments extends beyond this set. Novel polymer blends, degraded plastics with altered spectral signatures, and composite materials may introduce additional classification challenges that are not addressed by the current framework. The model’s ability to generalize to previously unseen plastic types or to detect anomalous materials therefore requires further investigation. One possible extension would be to introduce an unknown class for instances in which the model does not confidently match any of the 13 defined classes (12 polymer classes and 1 meat class). Third, the experiments were conducted under controlled laboratory conditions using carefully prepared samples. In real-world deployment, variability in lighting conditions, surface moisture, temperature, and mechanical vibration may affect classification performance. Although the calibration procedure accounts for some instrumental variation, the robustness of the model under diverse operating conditions should be validated through comprehensive field testing. Finally, although the computational efficiency and practical throughput gains provided by CFLA and LRP are substantial, practical deployment at industrial line speeds may still require dedicated GPU hardware. Further optimization through model quantization, pruning, or hardware-specific acceleration could improve deployment flexibility, particularly for facilities with limited computational resources.

## 4. Conclusions

This study presents significant advancement in food safety technology through the development of a novel transformer-based framework for classifying FPOs found in poultry products using NIR HSI. The proposed architecture, which combines CFLA, parameter-efficient LRP, and patch-local mixed-axis 2D RoPE with learnable frequencies, achieved the best overall performance among the compared models, with an OA of 99.39%, an AA of 99.57%, and a Kappa coefficient of 99.31 across 12 polymer classes and 1 meat class. These results demonstrate that spatial–spectral transformer architectures, when carefully adapted to the characteristics of hyperspectral data and pixel-level classification, can provide highly reliable discrimination of diverse polymer contaminants on poultry meat. The consistent performance across diverse FPO types, from common packaging materials to industrial equipment components, validates the framework’s robustness and practical utility for contamination source traceability. Beyond classification accuracy, the proposed framework also demonstrated substantial efficiency gains. Relative to the baseline configuration without LRP and CFLA, the final model reduced MACs and FLOPs by approximately 83%, decreased the parameter count from 451.2 K to 352.9 K, and reduced model size from 1.72 MB to 1.35 MB. These architecture-level improvements also translated into practical inference benefits, with the final configuration achieving a throughput of 212,971.5 hyperspectral patch cubes/s and a 4.19× speedup over the baseline. The combined gains in predictive performance, architectural efficiency, and inference speed indicate that the proposed framework supports high-throughput inference for real-time monitoring in industrial food processing environments. The proposed framework also has broader implications beyond the immediate application studied here. In particular, CFLA and patch-local mixed-axis 2D RoPE offer transferable design principles for other hyperspectral imaging tasks in which efficient spatial–spectral modeling is essential. At the same time, several directions remain for future work, including the identification of smaller plastic fragments (2 mm or smaller microplastic fragments), the incorporation of multi-scale or object-level constraints, improved handling of previously unseen polymer types, and field validation under realistic operating conditions. Overall, this work establishes a strong benchmark for transformer-based FPO classification and provides a practical foundation for contamination monitoring, source traceability, and preventive quality control in poultry processing.

## Figures and Tables

**Table 1:** Classification results of the compared CNN- and transformer-based models in terms of overall accuracy (OA), average accuracy (AA), Cohen’s Kappa coefficient (κ), and class-wise recall (%). All values are reported as mean ± standard deviation over 10 runs. The bold values represent the best results across all models.

Metrics	2D-CNN	3D-CNN	SpectralFormer	HiT	LSGA	Ours
OA (%)	79.61 ± 0.67	86.81 ± 0.61	94.94 ± 0.69	90.04 ± 0.64	96.36 ± 0.82	**99.39 ± 0.11**
AA (%)	85.90 ± 0.63	91.55 ± 0.61	92.38 ± 0.56	89.55 ± 0.71	97.09 ± 0.64	**99.57 ± 0.15**
κ (×100)	76.00 ± 0.31	83.86 ± 0.34	93.52 ± 0.46	88.56 ± 0.61	95.30 ± 0.86	**99.31 ± 0.18**
**Class**						
Fillet	63.63 ± 0.72	76.73 ± 0.74	97.29 ± 0.48	94.15 ± 0.96	94.94 ± 0.77	**98.59 ± 0.15**
ABS	70.32 ± 0.45	94.10 ± 0.35	99.27 ± 0.56	88.68 ± 0.56	98.87 ± 0.86	**99.97 ± 0.14**
FAB	92.74 ± 0.44	94.53 ± 0.61	83.97 ± 0.56	92.72 ± 0.72	99.72 ± 0.36	**99.95 ± 0.17**
HDPE	93.75 ± 0.94	95.29 ± 0.38	64.66 ± 0.37	66.43 ± 0.83	95.59 ± 0.67	**99.78 ± 0.16**
LDPE	97.86 ± 0.43	**99.87 ± 0.39**	76.70 ± 0.38	89.87 ± 0.44	99.41 ± 0.94	99.19 ± 0.19
NYL	99.64 ± 0.50	**99.77 ± 0.56**	94.38 ± 0.72	92.95 ± 0.95	99.27 ± 0.47	99.64 ± 0.15
PET	76.01 ± 0.52	78.39 ± 0.35	98.95 ± 0.68	98.01 ± 0.70	92.23 ± 0.60	**99.74 ± 0.12**
PP	74.46 ± 0.62	72.77 ± 0.87	92.45 ± 0.40	82.10 ± 0.84	92.74 ± 0.43	**99.08 ± 0.17**
PS	61.96 ± 0.92	86.81 ± 0.93	97.51 ± 0.70	92.45 ± 0.37	93.32 ± 0.94	**98.67 ± 0.16**
PUR	99.33 ± 0.77	98.76 ± 0.84	98.55 ± 0.49	87.55 ± 0.78	98.90 ± 0.45	**99.93 ± 0.11**
PVC	95.81 ± 0.86	97.23 ± 0.48	99.78 ± 0.31	98.38 ± 0.59	98.31 ± 0.42	**99.95 ± 0.10**
RUB	92.39 ± 0.68	96.63 ± 0.54	98.67 ± 0.95	80.95 ± 0.76	99.31 ± 0.39	**99.94 ± 0.14**
TEF	98.81 ± 0.31	99.33 ± 0.94	98.77 ± 0.72	99.95 ± 0.78	99.54 ± 0.96	**99.98 ± 0.19**

**Table 2:** Per-class precision (%), recall (%), and F1-score (%) of the proposed model. All values are reported as mean ± standard deviation over 10 runs.

Metrics	Fillet	ABS	FAB	HDPE	LDPE	NYL	PET	PP	PS	PUR	PVC	RUB	TEF
**Precision**	99.86 ± 0.18	98.75 ± 0.17	99.73 ± 0.19	99.07 ± 0.18	99.58 ± 0.20	98.05 ± 0.16	99.67 ± 0.18	98.45 ± 0.17	99.13 ± 0.19	99.70 ± 0.18	99.74 ± 0.20	99.65 ± 0.17	99.69 ± 0.17
**Recall**	98.59 ± 0.15	99.97 ± 0.14	99.95 ± 0.17	99.78 ± 0.16	99.19 ± 0.19	99.64 ± 0.15	99.74 ± 0.12	99.08 ± 0.17	98.67 ± 0.16	99.93 ± 0.11	99.95 ± 0.10	99.94 ± 0.14	99.98 ± 0.19
**F1-score**	99.22 ± 0.12	99.36 ± 0.11	99.84 ± 0.13	99.42 ± 0.12	99.38 ± 0.14	98.84 ± 0.11	99.70 ± 0.11	98.76 ± 0.12	98.90 ± 0.12	99.81 ± 0.11	99.84 ± 0.11	99.79 ± 0.11	99.83 ± 0.13

**Table 3:** Ablation study of LRP and CFLA in terms of classification performance and architecture-level efficiency. Classification metrics are reported as mean ± standard deviation over 10 runs, whereas efficiency metrics are deterministic and are reported once. The bold values represent the best results across each metric.

Index	LRP	CFLA	Classification Metrics			Efficiency Metrics			
			OA (%)	AA (%)	κ (×100)	MACs (M)	FLOPs (M)	# Parameters (K)	Model Size (MB)
1	×	×	99.37 ± 0.18	99.56 ± 0.16	99.29 ± 0.21	22.51	45.03	451.2	1.72
2	×	✔	99.32 ± 0.29	99.49 ± 0.25	99.21 ± 0.26	6.55	13.11	451.2	1.72
3	✔	✔	**99.39 ± 0.11**	**99.57 ± 0.15**	**99.31 ± 0.18**	**3.80**	**7.60**	**352.9**	**1.35**

**Table 4:** Empirical inference efficiency of ablation configurations with and without LRP and CFLA at inference batch size of 1024. Latency per hyperspectral patch cube, throughput, and speedup were measured over 1000 timed runs after 50 warmup iterations. Speedup is computed relative to the baseline configuration without LRP and without CFLA. The bold values represent the best values across each metric.

Index	LRP	CFLA	Inference Metrics		
			Latency/Patch (ms)	Throughput (Patches/s)	Speedup (×)
1	×	×	0.0197 ± 0.0004	50,885.5 ± 919	1.00×
2	×	✔	0.0052 ± 0.0003	192,310.0 ± 938	3.78×
3	✔	✔	**0.0047 ± 0.0000**	**212,971.5 ± 834**	**4.19×**

**Table 5:** Comparison of positional encoding mechanisms on the test set in terms of overall accuracy (OA), average accuracy (AA), and Cohen’s Kappa coefficient (κ). All values are reported as mean ± standard deviation over 10 runs. The bold values represent the best values across each accuracy metric.

Position Embedding Mechanism	OA (%)	AA (%)	κ (×100)
APE	99.03 ± 0.18	99.18 ± 0.22	98.96 ± 0.16
RoPE 2D Mixed	**99.39 ± 0.11**	**99.57 ± 0.15**	**99.31 ± 0.18**

## Data Availability

The data presented in this study are available upon request from the corresponding author but are not publicly accessible without authorization from the USDA-ARS. The code will be made public upon publication.

## Abbreviations

The following abbreviations are used in this manuscript:

AA	Average Accuracy
ABS	Acrylonitrile Butadiene Styrene
APE	Absolute Positional Embedding
BN	Batch Normalization
CA	Class Accuracy
CFLA	Center-Focused Linear Attention
CM	Confusion Matrix
CNN	Convolutional Neural Network
DL	Deep Learning
DN	Digital Number
ENVI	Environment Visualizing Imagery
FAB	Fabric
FDA	Food and Drug Administration
FLOP	Floating-Point Operation
FM	Foreign Material
FPO	Foreign Plastic Object
HDPE	High-Density Polyethylene
HSI	Hyperspectral Imaging
InGaAs	Indium Gallium Arsenide
Kappa	Cohen’s Kappa coefficient
LDPE	Low-Density Polyethylene
MAC	Multiply–Accumulate Operation
MLP	Multi-Layer Perceptron
NIR	Near-Infrared
NYL	Nylon
OA	Overall Accuracy
PCA	Principal Component Analysis
PE	Polyethylene
PET	Polyethylene Terephthalate
PLS-DA	Partial Least-Squares Discriminant
PP	Polypropylene
PS	Polystyrene
PTFE	Polytetrafluoroethylene
PUR	Polyurethane
PVC	Polyvinyl Chloride
ReLU	Rectified Linear Unit
RGB	Red, Green, Blue
ROI	Region of Interest
RoPE	Rotary Position Embedding
RUB	Rubber
SPA	Successive Projection Algorithm
SVM	Support Vector Machine
TEF	Teflon
USDA	U.S. Department of Agriculture

## Appendix A. 2D RoPE Implementation

To initialize the learnable frequencies in each layer, we define the following:

(A1) $$\begin{array}{cc} M_{k} & =\frac{1}{\theta_{\mathrm{base}}^{k/(d/4)}},\;k\in \left\{0,\ldots ,d/4-1\right\},\\ \alpha & ∼U[0,2\pi ],\\ {\tilde{\theta }}^{x} & =M_{k}\cdot \left[\cos\left(\alpha \right),\cos\left(\alpha +\pi /2\right)\right],\\ {\tilde{\theta }}^{y} & =M_{k}\cdot \left[\sin\left(\alpha \right),\sin\left(\alpha +\pi /2\right)\right], \end{array}$$

where *d* denotes the dimensionality of the RoPE-applied feature subspace.

For a patch of size P×P, token positions $\left(x_{i},y_{i}\right)$ are assigned as follows:

(A2) $$\begin{array}{cc} x_{i} & =i\;\mathrm{mod}\;P,\;i\in \{0,\ldots ,P^{2}-1\},\\ y_{i} & =\lfloor i/P\rfloor . \end{array}$$

These positions define the mixed-axis rotation phase:

(A3) $$\begin{array}{cc} \Phi_{i} & =x_{i}\cdot {\tilde{\theta }}^{x}+y_{i}\cdot {\tilde{\theta }}^{y},\\ R_{i} & =e^{\;i\Phi_{i}}. \end{array}$$

Because CFLA uses only the center token as the query, the RoPE transformation is applied to the center query vector and to all key vectors as follows:

(A4) ic=⌊P2/2⌋,qc′=Re(qc·Ric),K′=Re{ki·Ri}i=1P2.
